# Plants target gut microbes to reduce insect herbivore damage

**DOI:** 10.1073/pnas.2308568120

**Published:** 2023-06-28

**Authors:** Hannah M. McMillan

**Affiliations:** ^a^Department of Biology, Duke University, Durham, NC 27708

**Fig. 1.**
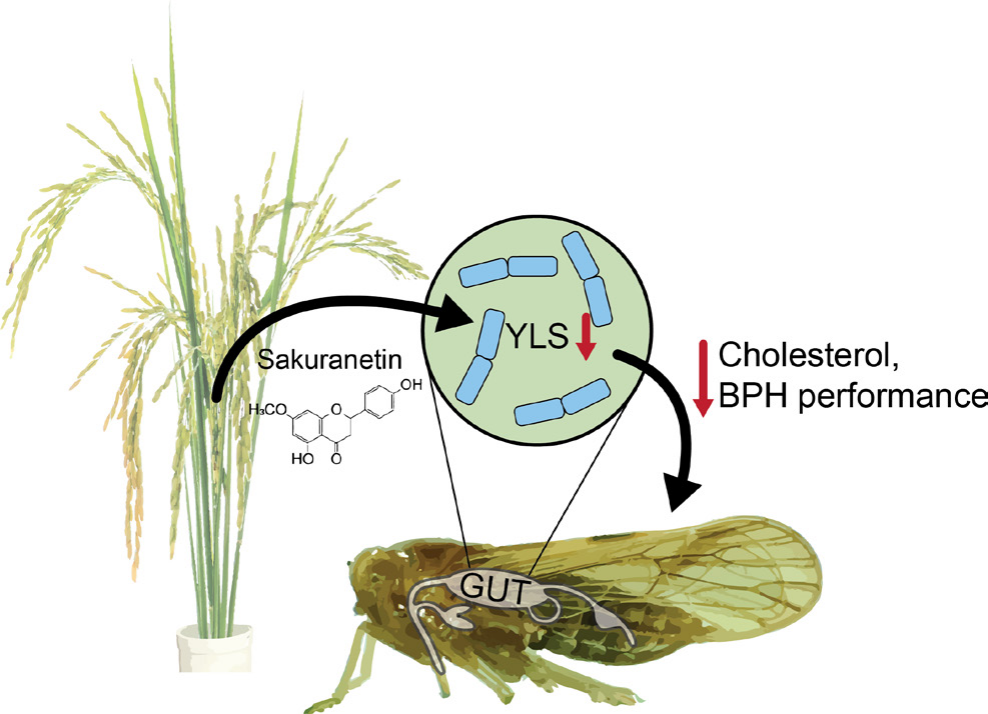
Proposed model for sakuranetin defense mechanism. BPH attack elicits sakuranetin production in rice. Sakuranetin is ingested during BPH feeding and subsequently reduces the number of YLS in the BPH gut. Decreased YLS abundance reduces BPH performance by limiting cholesterol production, resulting in improved plant health.

Plants must constantly identify and respond to a variety of abiotic and biotic stress, and they must do so in ways that balance defense and stress responses with growth and reproduction. In agricultural settings, understanding the mechanisms behind plant growth and stress trade-offs could facilitate identification and design of safe, effective treatments to improve plant performance, crop yield, and plant health. With respect to biotic threats such as bacteria, fungi, and insect herbivores, plants often produce specialized metabolites that act either directly to neutralize the attack or indirectly by activating various local and systemic responses (1). Many defensive plant metabolites have been identified; however, their mechanism of action is often unknown or poorly understood, especially for insect herbivores (2). Intriguingly, microbial endosymbionts can play important roles in plant–insect interactions (3–5), suggesting that previous research may have overlooked important multi-level interaction networks that determine the outcome of herbivore attack. In PNAS, Liu et al. explore one such network of interactions and show that a rice defense flavonoid, sakuranetin, targets and reduces the abundance of yeast-like beneficial endosymbionts in brown planthoppers (BPHs), thereby reducing BPH performance and improving plant health (Fig. 1) (6).

In PNAS, Liu et al. explore one such network of interactions and show that a rice defense flavonoid, sakuranetin, targets and reduces the abundance of yeast-like beneficial endosymbionts in brown planthoppers (BPHs), thereby reducing BPH performance and improving plant health (6).

The brown planthopper *Nilaparvata lugens* is a destructive phloem-feeding herbivore that causes hundreds of millions of US dollars in rice crop losses annually (7, 8). Indeed, BPH damage leads to 20 to 80% yield loss, and in areas with heavy infestations, BPH damage can result in near-total crop failure (9). While chemical insecticides effectively control BPHs, application poses significant risks for human health and eliminates natural BPH predators. Without predators, BPH populations increase unchecked, which can lead to greater plant damage during outbreaks and in subsequent years (7, 9). Further, BPHs display high levels of resistance to many insecticides (7). Safer, more effective BPH treatments are clearly needed to address these issues.

The main driver of plant immune responses to insect herbivores is the phytohormone jasmonate, which is induced upon insect attack and regulates the biosynthesis of many downstream defense proteins and metabolites (10). Some of these metabolites, such as the alkaloid nicotine and volatile terpenes, have broad impacts on insect pests including neuromuscular impairment and repellent effects (11, 12). In response to BPH attack, jasmonate signaling in rice plants regulates genes in the phenylpropanoid pathway, strongly increasing expression of flavonoid biosynthetic genes (13). This signaling network increases levels of the defense metabolite sakuranetin and subsequently reduces BPH performance; however, the precise mechanism of action for sakuranetin is unknown (13).

One unexplored target for plant defense metabolites like sakuranetin is the insect microbiome (14). Microbial endosymbionts play many beneficial roles for their insect hosts, including essential roles in growth, development, reproduction, stress resilience, and insect–plant interactions (5). In BPHs, yeast-like symbionts (YLSs) are the most abundant endosymbionts, are transmitted via eggs, and are found in every developmental stage of BPHs (15, 16). Critically, YLSs provide essential amino acids and sterol precursors for BPHs and aid in nitrogen recycling (17–19). These microbes play such an important role that depleting YLSs by heat treatment reduces growth of BPHs, resulting in lower body weight and delayed emergence (18). In light of previous data showing that sakuranetin has broad antifungal activity (20), Liu et al. hypothesized that this defense metabolite may reduce BPH performance by targeting YLSs.

As expected, Liu et al. show that BPH nymph survival decreased when fed an artificial diet with sakuranetin (6). Further, when allowed to feed on sakuranetin-deficient rice plants, BPH developmental rates were shorter, more eggs were produced, and hatching rates increased, confirming that sakuranetin decreased BPH performance (6). To probe whether sakuranetin decreased BPH performance via an effect on YLSs, Liu et al. used fungal ITS sequencing to characterize the BPH microbiome (6). Indeed, the two most abundant fungal taxa, *Cordyceps* and *Candida*, were enriched in BPHs that were fed on sakuranetin-deficient rice (6). Of note, the *Cordyceps* genus contains Ascomycetes YLSs, which are the dominant fungal endosymbionts in BPHs (15). Microscopy and qPCR experiments confirmed that in addition to altering the relative abundance of fungal endosymbionts, sakuranetin reduced the overall number of YLSs in BPHs (6).

These results provide compelling evidence that sakuranetin impacts the BPH microbiome and specifically reduces the abundance of YLSs; however, reduced YLS abundance could be an indirect result of a direct interaction between BPH and sakuranetin. Unfortunately, YLSs cannot be cultured outside of BPHs, which prohibits studying the direct impact of sakuranetin on YLSs in vitro (15). To circumvent this challenge and limit the influence of BPH responses, Liu et al. cleverly tested direct anti-YLS activity of sakuranetin using fresh BPH egg homogenate and showed that sakuranetin significantly reduced YLSs in egg homogenate and in fresh, intact BPH eggs (6). These results suggest that sakuranetin acts directly on the YLSs rather than on BPHs (6).

As shown previously, depleting YLSs has a significant negative impact on BPH performance and results in lower body weight and delayed emergence (18). In part, these effects are seen because YLSs provide BPHs with a basic sterol precursor that is required for cholesterol biosynthesis (17, 19). Indeed, Liu et al. show that when YLSs are depleted by sakuranetin, BPHs have significantly lower cholesterol levels than BPHs fed on sakuranetin-deficient rice that retain their YLSs (6). Increased cholesterol in BPHs with YLSs likely contributes to improved BPH performance and suggests a possible mechanism for plant defense in which the plant metabolite indirectly targets the insect herbivore by disrupting the insect microbiome (Fig. 1). Further, this finding highlights the importance of multilevel, interspecies interactions in determining outcomes for plant health.

These results reveal a new area for study when considering how plants defend against herbivore attack and raise a variety of questions to be considered in future mechanistic studies. It is curious that a plant could develop a defense response against a microbe it never physically encounters. One may consider whether insect gut microbes secrete signals that travel through the insect mouthparts during feeding and, in turn, elicit plant defense responses. Conversely, it has been shown that microbes present in herbivore saliva can suppress jasmonate signaling, dampening plant defense responses and benefiting the insect host (5, 21). Perhaps it is also possible that herbivores recruit specific gut microbes that secrete signals to suppress plant responses. This new line of questioning may also reveal novel approaches to control plant pathogens that are spread by insect vectors, such as *Candidatus Liberibacter asiaticus* that causes the devastating citrus greening disease. In this scenario, crops could be engineered to express metabolites similar to sakuranetin for delivery as the insect feeds to its gut microbes or those present in herbivore mouthparts. As a result, this approach may eliminate microbial pathogens before they are transmitted to the plant host and cause disease. With this study, Liu et al. have opened the door to explore many exciting new directions in plant–insect interactions with the potential to unveil effective new agricultural control measures for both herbivore pests and insect-vectored microbial plant pathogens.
